# Effect of Cross-Linking and Enzymatic Hydrolysis Composite Modification on the Properties of Rice Starches

**DOI:** 10.3390/molecules17078136

**Published:** 2012-07-06

**Authors:** Huaxi Xiao, Qinlu Lin, Gao-Qiang Liu

**Affiliations:** 1 National Engineering Laboratory for Rice and By-product Further Processing, Central South University of Forestry & Technology, Changsha 410004, China; Email: xiaoxijingjing@163.com (H.X.); lql0403@yahoo.com.cn (Q.L.); 2 Faculty of Food Science and Engineering, Central South University of Forestry & Technology, Changsha 410004, China; 3 College of Life Science and Technology, Central South University of Forestry & Technology, Changsha 410004, China

**Keywords:** rice starch, cross-linking, enzymatic hydrolysis, hydrolyzed rice starch, cross-linked rice starch

## Abstract

Native rice starch lacks the versatility necessary to function adequately under rigorous industrial processing, so modified starches are needed to meet the functional properties required in food products. This work investigated the impact of enzymatic hydrolysis and cross-linking composite modification on the properties of rice starches. Rice starch was cross-linked with epichlorohydrin (EPI) with different concentrations (0.5%, 0.7%, 0.9% *w/w*, on a dry starch basis), affording cross-linked rice starches with the three different levels of cross-linking that were named R_1_, R_2_, and R_3_, respectively. The cross-linked rice starches were hydrolyzed by α-amylase and native, hydrolyzed, and hydrolyzed cross-linked rice starches were comparatively studied. It was found that hydrolyzed cross-linked rice starches showed a lower the degree of amylase hydrolysis compared with hydrolyzed rice starch. The higher the degree of cross-linking, the higher the capacity to resist enzyme hydrolysis. Hydrolyzed cross-linked rice starches further increased the adsorptive capacities of starches for liquids and decreased the trend of retrogradation, and it also strengthened the capacity to resist shear compared to native and hydrolyzed rice starches.

## 1. Introduction

Rice is one of the most important cereals and commercially more than two thousand varieties of rice are grown throughout the World. Native rice starches have many properties that differ from those produced from other plant sources. These inherent characteristics can be exploited to produce various types of starch-based food products, but native rice starch lacks the versatility necessary to function adequately under rigorous industrial processing. Thus, a variety of ways have been developed to modify starch to provide the desired stability and tolerance toward a wide range of processing techniques. The native starch can readily be modified by physical, chemical or enzymatic processes to afford products with improved starch functionality and consequently these products find diverse applications [1]. 

Starch was treated by enzyme (α-amylase, glucoamylase or pullulanase) at a temperature below the pasting temperature of starch. Enzymatically hydrolyzed starch is a natural susbtance which has strong adsorbent capability and retarded retrogradation. However, hydrolyzed starch showed lower capacity to resist shear compared with native starch. Cross-linked starches constitute a major class of modified starches. Cross-linking of starch by reaction with bi- or polyfunctional reagents is widely used in numerous industrial applications such as preparation of wet-rub-resistant starch paper coatings, permanent textile sizes and water-resistant adhesives [2]. Cross-linking reinforces the already present hydrogen bonds in the granules with new covalent bonds. As a result, cross-linked starch is more resistant to acid, enzymatic hydrolysis, heat and shearing than native starch and is thus suitable for applications in food industry. Cross-linking of starch with epichlorohydrin (EPI) is the most common method used in polysaccharide chemistry [3,4]. Up until now there has been a shortage of data on the cross-linking and hydrolysis composite modification of rice starches. The objective of this study was to evaluate the impact of enzymatic hydrolysis and cross-linking composite modification on the properties of rice starches.

## 2. Results and Discussion

### 2.1. Degree of Cross-Linking

The degree of cross-linking of the cross-linked rice starch was determined from the RVA viscosity values and the results are presented in Table 1. It was observed that the degree of cross-linking increased with the increasing concentration of EPI. In a previous study on physicochemical properties of chemically modified starches from different botanical origin [5], at a lower level of EPI (0.3%), the degree of cross-linking was not detectable by the viscosity method used. The degree of cross-linking of starch cross-linked with lower level of EPI (0.3%) was not great enough to restrict the swelling of the granules and these exhibited higher peak viscosity values than that of native starch [6]. However, when the amount of EPI was increased to 0.5%, the degree of cross-linking of starch began to be determined. At 0.9% EPI level, nearly 72.4% cross-linking was obtained. Higher level of cross-link of starch retarded the swelling of granules, resulting in a lower peak viscosity.

**Table 1:** Degrees of cross-linking of rice starch modified with epichlorohydrin (EPI).

Samples	Concentration of EPI (%)	Degree of cross-linking (%)
R_1_	0.5	18.71 ± 0.11c
R_2_	0.7	31.62 ± 0.23b
R_3_	0.9	72.41 ± 0.38a

The values are average ± standard deviation, n = 3. Values followed by the same letter in the same column are not significantly different (*p* > 0.05). R_1_, R_2_, R_3_, the cross-linked rice starch produced with 0.5%, 0.7%, 0.9% of EPI, respectively.

### 2.2. Hydrolysis of Starches

The hydrolysis of native and cross-linked rice starches with different degrees of cross-linking is shown in Table 2. It was apparent that native rice starch was hydrolyzed more effectively than cross-linked rice starch, and the total amount of hydrolysis of native rice starch was 53.2%. However, with increasing degree of cross-linking, the total amount of hydrolysis of cross-linked rice starch reduced gradually from 40.2% to 11.4%, suggesting the cross-linked rice starch was more resistant to hydrolysis in comparison to native rice starch. According to Oates, the less organized amorphous rings are susceptible to enzyme attack, whereas the crystalline lamellae are resistant to enzyme activity [7]. A study showed that a higher degree of hydrolysis for enzyme-hydrolyzed starch may be due to extensive penetration by the enzyme, which was able to hydrolyze the amorphous and crystalline lamellae deep in the granule [8]. Most chemical modifications firstly occur in amorphous regions, presumably because amorphous regions are more accessible to the modifying reagent. Shiftan *et al*. reported that EPI cross-linking was not homogeneous and was concentrated in the non-crystalline domain of starch granules [9]. Jane *et al*. found that cross-linking of starch chains occurred mainly in amylopectin and some amylose molecules were cross-linked to amylopectin [10]. The cross-linking markedly reinforced the structure of starch granule by introducing intermolecular bridges between starch chains in both amorphous and crystalline regions. The cross-linking reduced the accessibility of the enzyme to the starch, so the higher the degree of cross-linking, the stronger the capacity to resist enzyme hydrolysis.

**Table 2:** Degrees of hydrolysis of native and cross-linked rice starches with different degrees of cross-linking at 40 °C for 24 h.

Rice starches	Total amount of hydrolyzed material (%)
Hydrolyzed	53.23 ± 0.71a
Hydrolyzed-R_1_	40.21 ± 0.52b
Hydrolyzed-R_2_	29.80 ± 0.37c
Hydrolyzed-R_3_	11.44 ± 0.14d

The values are average ± standard deviation, n = 3. Values followed by the same letter (a,b,c or d) in the same column are not significantly different (*p* > 0.05). R_1_, R_2_, R_3_, the cross-linked rice starch produced with 0.5%, 0.7%, 0.9% of EPI, respectively.

### 2.3. Adsorptive Capacity of Starches for Liquids

The adsorptive capacities of native, hydrolyzed, and hydrolyzed cross-linked rice starches for water and oil are presented in Table 3. The modified starches exhibited a decrease in the adsorptive capacities of starches for liquids with increasing degree of cross-linking. The adsorptive capacities of hydrolyzed cross-linked rice starches ranged from 3.84 to 2.42 for water and from 2.14 to 1.43 for oil, respectively, however, they all were higher than those of native and hydrolyzed rice starches (Table 3).

**Table 3:** Adsorption capacity of native, hydrolyzed, and hydrolyzed cross-linked rice starches for liquids at room temperature.

Rice starch	Adsorptive capacity (g/g sample)	
	Water	Oil
Native	1.61 ± 0.03e	0.66 ± 0.01e
Hydrolyzed	1.98 ± 0.04d	0.96 ± 0.01d
Hydrolyzed-R_1_	3.84 ± 0.06a	2.14 ± 0.06a
Hydrolyzed-R_2_	3.01 ± 0.05b	1.81 ± 0.04b
Hydrolyzed-R_3_	2.42 ± 0.08c	1.43 ± 0.03c

The values are average ± standard deviation, n = 3. Values followed by the same letter in the same column are not significantly different (*p* > 0.05). R_1_, R_2_, R_3_, the cross-linked rice starch produced with 0.5%, 0.7%, 0.9% of EPI, respectively.

It is well known that α-amylase can drill and widen the pinholes in starch granules. Helbert *et al*. proposed the concept of “centripetal” hydrolysis (surface-to-core directed) and “centrifugal” hydrolysis (involving regions and layers) of native granules by α-amylase [11]. The studies [11,12] found that the enzyme first randomly diffuses onto the surface of granules. Hydrolysis then starts at these points, proceeds radially toward the center (centripetal) and results in the formation of a pore and ultimately a channel to the granule core. Finally, the enzyme becomes trapped within the granule and causes diffusion-regulated local hydrolysis, which gradually spreads (centrifugal). The extensive penetration by the enzyme caused the pores on or in starch granules to cave in. The cross-linking retarded the caving process and kept the integrity of pores by strengthening the structure of starch granules, which resulted in the increase in the adsorptive capacities of starches for liquids.

### 2.4. Pasting Properties

The RVA viscosity characteristics of native and modified starches are shown in Table 4. It can be found that the pasting temperatures of hydrolyzed starches were lower, however those of hydrolyzed cross-linked starches were higher in comparison to native starch and increased with the increasing degree of cross-linking. The peak viscosity and setback value of hydrolyzed and hydrolyzed cross-linked starches were lower compared with the native ones and showed a gradual decrease with the increasing degree of cross-linking. Hydrolysis destroyed the structure of native starch, however cross-linking strengthened the bonding between starch chains and increased the resistance of the granules to swelling, leading to lower peak viscosity. Hydrolyzed cross-linked starches showed lower breakdown values, which could be attributed to the strengthening of the swollen granules against breakage under conditions of high temperature and shear [6]. The breakdown value of hydrolyzed starch was lower than that of native starch, which could be due to the destroyed structure by amylase, which indicated hydrolysis decreased the resistance to shear. Setback viscosity is determined by the reassociation of solubilized starch polymers and insoluble granular fragments during cooling. The setback value of hydrolyzed and hydrolyzed cross-linked starches were lower compared with native starch, and the setback value of hydrolyzed cross-linked starch gradualy decreased with the increasing degree of cross-linking. Jyothl *et al.* [6] indicated that the setback value decreased at higher levels of cross-linking. The bonding force between molecules of hydrolyzed starch by the combination of hydrogen bonds was decreased due to the enzyme hydrolysis, which changes the order-disorder structure in starch molecules. When ordered arrays of starch molecules were disrupted, the reassociation of starch molecules was inhibited. These results suggested that enzyme hydrolysis and hydrolyzed cross-linking could effectively inhibit the retrogradation of rice starch.

**Table 4:** Pasting properties of native, hydrolyzed, and hydrolyzed cross-linked rice starches.

Rice starch	Pasting temp (°C)	Peak Visco (cP)	Breakdown (cP)	Setback (cP)
Native	78.9 ± 0.7	3186.2 ± 37.1	1478.1 ± 15.2	1754.3 ± 18.1
Hydrolyzed	75.2 ± 0.5	2738.3 ± 31.2	1563.1 ± 16.4	1104.1 ± 10.2
Hydrolyzed-R_1_	79.1 ± 0.8	2142.2 ± 23.6	874.2 ± 10.3	768.2 ± 6.4
Hydrolyzed-R_2_	79.8 ± 0.7	1649.1 ± 17.5	821.3 ± 9.5	705.1 ± 5.8
Hydrolyzed-R_3_	80.3 ± 0.9	1173.3 ± 11.4	783.4 ± 8.7	652.2 ± 4.9

The values are average ± standard deviation, n = 3. Values followed by the same letter in the same column are not significantly different (*p* > 0.05). R_1_, R_2_, R_3_, the cross-linked rice starch produced with 0.5%, 0.7%, 0.9% of EPI, respectively.

### 2.5. Thermal Properties

The transition temperatures and the enthalpy values of gelatinization and retrogradation of native, hydrolyzed, and hydrolyzed cross-linked rice starches are presented in Table 5. It can be seen that the transition temperatures and the enthalpy values of gelatinization of hydrolyzed cross-linked rice starches were higher in comparison to those of native and hydrolyzed rice starches and increased with the increasing degree of cross-linking, whereas the transition temperatures and the enthalpy values of gelatinization of hydrolyzed rice starch were the lowest. The decrease in transition temperatures and the enthalpy values of gelatinization for hydrolyzed starch was due to attacked amylose in the amorphous region influenced by amylase. Cooke and Gidley [13] have shown that Δ*H*_gel_ values primarily reflect the loss of double helical order rather than loss of crystalline register. The decrease in transition temperatures and Δ*H*_gel_ values of the hydrolyzed starch after amylase treatment suggested that some of the double helices present in the crystalline and non-crystalline regions of the granule may have disrupted under the conditions prevailing during amylase treatment. The decrease in Δ*H*_gel_ values indicated that hydrolyzed starch required less thermal energy for gelatinization compared to the native starches. Heat of gelatinization reflects the energy required for disrupting the starch granule structure and since cross-linking reinforces the structure of starch granules, more heat will be required for gelatinization.

**Table 5:** DSC characteristics of native, hydrolyzed, and hydrolyzed cross-linked rice starches.

Rice starch	Gelatinization (°C)			Retrogradation (°C)		
	*T*_o_	*T*_p_	Δ*H*_gel_ (J/g)	*T*_o_	*T*_p_	Δ*H*_ret_ (J/g)
Native	64.82± 0.51d	71.63 ± 1.01d	7.81 ± 0.22d	52.14 ± 0.55d	55.42 ± 0.43d	2.81 ± 0.11a
Hydrolyzed	61.54 ± 0.62e	70.41 ± 0.84e	5.30 ± 0.14e	48.61 ± 0.54e	52.34 ± 0.40e	1.21 ± 0.09b
Hydrolyzed-R_1_	70.21 ± 0.83c	80.12 ± 0.95c	8.53 ± 0.31c	57.63 ± 0.58c	59.72 ± 0.52c	0.81 ± 0.08c
Hydrolyzed-R_2_	75.50 ± 0.94b	87.20 ± 1.10b	9.21 ± 0.36b	62.30 ± 0.61b	66.51 ± 0.57b	0.51 ± 0.07d
Hydrolyzed-R_3_	79.82 ± 0.88a	91.43 ± 1.23a	10.32 ± 0.41a	66.51 ± 0.67a	68.83 ± 0.60a	0.11 ± 0.02e

The values are average ± standard deviation, n = 3. Values followed by the same letter in the same column are not significantly different (*p* > 0.05). R_1_, R_2_, R_3_, the cross-linked rice starch produced with 0.5%, 0.7%, 0.9% of EPI, respectively.

The molecular interaction (hydrogen bonding between starch chains) after cooling of the gelatinized starch paste has been called retrogradation [14]. The enthalpy values of the retrograded starch reflect the melting of the crystallites formed by the association between adjacent double helices during gel storage [15]. Retrogradation properties of starch are indirectly influenced by structural arrangements of starch chains within the amorphous and crystalline regions of the ungelatinized granule [16]. The retrograded starches showed lower transition temperature and enthalpy in the second run heating DSC than native starches in the first run heating DSC. These results suggest that retrogradation results in reassociation of the gelatinized starch molecules, but in less ordered form and hence less perfect or stable forms than those existing in the native starch granules [17]. It can be seen that hydrolyzed cross-linked rice starches showed a lower tendency of retrogradation than did native and hydrolyzed rice starches. This result indicated a lower degree of starch molecule reassociation in the hydrolyzed cross-linked rice starches. The enthalpy values of the retrograded hydrolyzed rice starches were significantly lower than those of their native rice starches. Retrograded starch forms strong hydrogen bonds between the molecules and completes a cement structure in amorphous regions. The bonding force between molecules of hydrolyzed rice starch by the combination of hydrogen bonds was decreased due to the enzyme hydrolysis, which inhibits the retrogradation of hydrolyzed starch. It is shown in Table 5 that the retrogradation of hydrolyzed cross-linked rice starch decreased with the increasing degree of cross-linking, so it could be suggested that the restricted mobility of the high cross-linked amylose and amylopectin branches could retard the reassociation of the starch chains and inhibited the recrystallization.

### 2.6. Flowing Characteristics

**Figure 1:**
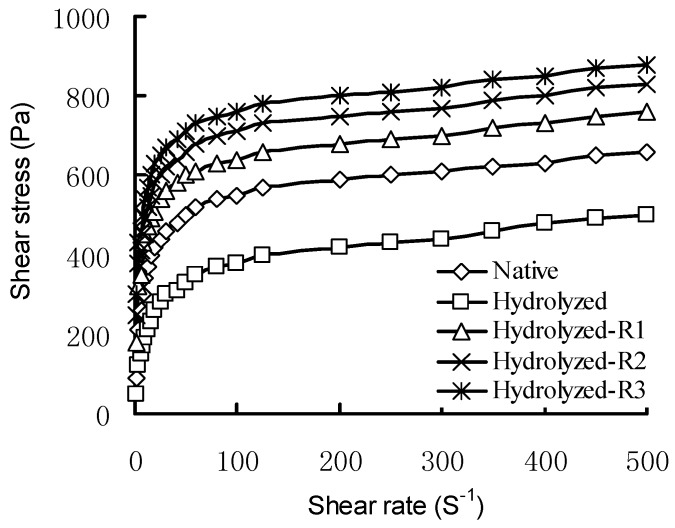
Shear stress as a function of shear rate for native, hydrolyzed, and hydrolyzed cross-linked rice starches.

Previous results indicated that rice starches were pseudoplastic and shear thinning liquids. As was shown by the flow curves in Figure 1, shear stress of all native and modified starches increased with increasing shear rate. At the same shear rate, hydrolyzed rice starch showed the lowest shear stress, followed by native rice starch, whereas hydrolyzed cross-linked rice starches showed higher shear stress compared with native and hydrolyzed rice starches, and the shear stress increased with the increasing degree of cross-linking. High values of shear stress pointed to a high stability of the structure of the starch [18]. According to Gibinski *et al*., cross-linked starch had the most stable structure and the strong ability to resistant to shear, the structure of hydrolyzed starch was weakened and it lost the ability to be resistant to shear [18]. Strengthening the bonding between starch chains through cross-links resulted in stable structures. However, the partially degraded network was not resistant to shear and could not maintain the integrity of starch granules after hydrolysis, resulting in a decrease in shear stress. The cross-linking improved the resistance to shear of hydrolyzed starch.

## 3. Experimental

### 3.1. Materials

Native unmodified rice starch was purchased from Puer Yongji Biological Technology Co. Ltd. (Kunming, China). The contents of moisture, ash, and protein in this rice starch are 11.2%, 0.15%, and 0.50%, respectively, while the amylose content is 19.5% (data from Puer Biological Technology Co. Ltd., Kunming, China). EPI and α-amylase (activity 4,000 IU/mg) were purchased from NongHe Co. Ltd. (Changsha, China). Here, IU/mg refers to 1 mg enzyme solution acting on 2.5% (*w/v*) raw starch in a buffer solution (pH 3.4, 0.2 mol/L Na_2_HPO_4_, 0.1 mol/L citric acid) and releasing 1 mg glucose in 1 h at 40 °C. All chemicals and reagents used in this study were of analytical grade. 

### 3.2. Preparation of Cross-Linked Starch

Cross-linking reaction of rice starch was performed by suspending the starch (100 g, dry basis) in distilled water (150 mL) with added NaCl (3 g). The starch suspension was continuously stirred at room temperature. After adjusting to pH 10.0 with 1 mol/L NaOH, EPI with different concentrations (0.5%, 0.7%, 0.9% *w/w*, on a dry starch basis) was added directly to the slurry with stirring at 25 °C for 3 h, then the pH was adjusted to 6.0–6.5 with 0.2 mol/L HCl and the cross-linked rice starch was isolated by centrifugation (3,000 ×g, 15 min). After washing with distilled water, the sediment was dried at 45 °C for 48 h in a vacuum oven. The cross-linked rice starches produced with three levels of cross-linking were named R_1_, R_2_, and R_3_, respectively.

### 3.3. Degree of Cross-Linking

The degree of cross-linking of the cross-linked starches was determined from the viscosity values, according to the method of Chatakanonda *et al*. [19]. The peak viscosities of the starch samples were recorded using a Rapid Visco Analyser (RVA super 4, Newport Scientific, Sydney, Australia). The starch slurry (10% by weight) was heated from 50 to 95 °C at 12 °C/min at 160 rpm, and then held at 95 °C for 2 min. Afterwards the paste was cooled to 50 °C at 12 °C/min and finally kept at 50 °C for 2 min. The degree of cross-linking was calculated as follows: 


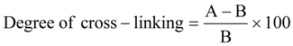
(1)

where A is the peak viscosity in RV units of the native starch and B is that of the cross-linked starch.

### 3.4. Hydrolysis of Starch by α-Amylase

Samples (100 g, in triplicate, accurately weighed) of native starch or cross-linked starch and α-amylase (5 g) were suspended in pH 3.4 buffer solution (125 mL, 0.2 mol/L Na_2_HPO_4_, 0.1 mol/L citric acid) and stirred continually for 24 h at 40 °C according to Yamada [20], then the solution was centrifuged, and the precipitate was washed with distilled water for four times to remove the enzyme, the product was dried at 45 °C for 48 h in a vacuum oven and ground. Pass through a 100-mesh sieve. Glucose was then determined according to Karkalas *et al*. [21].

### 3.5. Determination of Adsorptive Capacity for Liquids

The determination of adsorptive capacity of starch for liquids was performed according to the method of Nagai with a slight modification [22]. Starch (3 g) and liquid (10 mL, water or rape seed oil) were mixed and stirred for 30 min at room temperature, and then the mixture was filtrated by vacuum filtration, placed on a filter. After dripping of the liquids ceased, the weight of the liquid-impregnated sample was measured, from which the adsorptive capacity of the starch for the liquids was calculated as the weight of liquid-impregnated sample divided by the dry weigh of the sample. 

### 3.6. Pasting Properties

The pasting properties of native and modified starches were determined with the Rapid Visco Analyzer (RVA super 4, Newport Scientific, Sydney, Australia). The viscosities of the starches were recorded with starch suspensions (moisture content 12.0%, sample 3.00 g, water 25.00 mL). They underwent a controlled heating and cooling cycle under constant shear where it was held at 50 °C for 1 min. They were then heated from 50 to 95 °C at 5 °C/min and held at 95 °C for 2.7 min, cooled from 95 °C to 50 °C at 5 °C/min and held at 50 °C for 2 min. The initial speed of blending in 10 s was for 960 rpm, after that it was maintained at 160 rpm. Pasting parameters such as pasting temperature, peak viscosity, breakdown (peak viscosity-hot paste viscosity), final viscosity, setback (cold paste viscosity-peak viscosity) were recorded.

### 3.7. Differential Scanning Calorimetry (DSC)

The thermal characteristics of starches were measured using 61 DSC-Pyris Diamond (Perkin-Elmer Corp., Norwalk, CT, USA). Starch samples were weighed into aluminum DSC pans, and deionized water was added by micropipette to achieve a water-sample ratio of 2:1. The sample pans were sealed and allowed to stabilize at room temperature for 24 h before heating. Samples were heated at a rate of 10 °C/min from 25 to 100 °C, which used an empty pan as reference. The onset temperature (*T*_o_) and peak temperature (*T*_p_) were determined from the run heating DSC curves. Enthalpy of gelatinization (Δ*H*_gel_) was evaluated based on the area of the main endothermic peak. After the DSC run, gelatinized starch samples were stored at 4 °C for 7 day for retrogradation studies, these stored samples were scanned under the same conditions and the transition temperature and retrogradation enthalpy (Δ*H*_ret_) was determined from the second run heating. 

### 3.8. Measurement of Flow Behavior

Rive starch (8 g, dry basis, db) was suspended in distilled water (100 mL) and heated in boiling water bath for 25 min, followed by cooling to room temperature, the starch paste was obtained. Starch paste was put into the testing platform of dynamic rheometer (ARES, TA, Ltd., New Castle, DE, USA). Viscometry was performed using a controlled strain rheometer using parallel plate mould (40 mm diameter and 1 mm gap). After trimming off the over-loaded portion of samples around plates, the open side of samples was covered with a thin layer of silicon oil to prevent moisture loss. Shear stress with increasing shear rate (0–500/s) was obtained at 20 °C to characterize flow behavior. The applied measurement regime was as follows: rate of shear was increased from 0 to 500/s logarithmically within 600 s.

### 3.9. Statistical Analyses

The data reported in the tables were average of triplicate observations. Data obtained were analyzed by analysis of variance (ANOVA) using SPSS for windows version 13.0. Confidence interval of sample means was reported at the 95% confidence probability. Comparisons of means were made using least significant difference (LSD) and shortest significant ranges (SSR) at 5% significance level.

## 4. Conclusions

The amylase hydrolyzed rice starch has stronger adsorptive capacity and shows a lower retrogradation tendency compared with native rice starch, whereas hydrolyzed rice starch shows the weakest ability to resist shear in comparison to native rice starch. The cross-linking retarded the caving of pores caused by the extensive penetration of enzyme and kept the integrity of pores by strengthening the structure of starch granules, which resulted in an increase in the adsorptive capacities of starches for liquids. In the meantime hydrolyzed cross-linked rice starch further decreased the tendency of retrogradation and increased the ability to resist shear compared with hydrolyzed rice starch. The higher the degree of cross-linking, the stronger the resistance to shear and retrogradation. However, the capacity to resist enzyme hydrolysis of cross-linked starches increased with the increasing degree of cross-linking. It is concluded that hydrolyzed cross-linked rice starch overcomes the undesirable properties caused by retrogradation and low shear resistance. Considering starch is a common ingredient used widely in many food and non-food applications, and native rice starch lacks the versatility necessary to function adequately under industrial processing conditions, the hydrolyzed cross-linked rice starch could more effectively meet the functional property demands in food and non-food products 
